# Regional contributions to ventricular stroke volumes are affected on the left side, and not on the right in patients with pulmonary hypertension

**DOI:** 10.1186/1532-429X-17-S1-P294

**Published:** 2015-02-03

**Authors:** Ellen Ostenfeld, Sigurdur S Stephensen, Katarina Steding-Ehrenborg, Einar Heiberg, Håkan Arheden, Göran Rådegran, Johan Holm, Marcus Carlsson

**Affiliations:** 1 Cardiac MR group Lund, Dept. of Clinical Physiology, Lund University, Lund, Sweden; 2 Dept. of Pediatric Cardiology, Lund University Hospital, Lund University, Lund, Sweden; 3 Section for Heart Failure and Valvular Disease, Lund University Hospital, Lund University, Lund, Sweden

## Background

Right ventricular (RV) function is of prognostic value in patients with pulmonary hypertension (PH). Eighty percent of RV stroke volume (SV) comes from longitudinal ventricular function in a normal population, while longitudinal function accounts for 60 % of left ventricular (LV) SV. Radial function, consisting of septal and lateral function, accounts for the remaining contribution to SV. Longitudinal, septal and lateral changes in regional function has been seen as pressure is elevated on the right side.

The aim of this study was quantify the longitudinal, septal and lateral contributions to SV in patients with PH using cardiac magnetic resonance (CMR), and demonstrate if there is a relationship between pulmonary pressure and these contributors to SV.

## Methods

**Figure 1:**
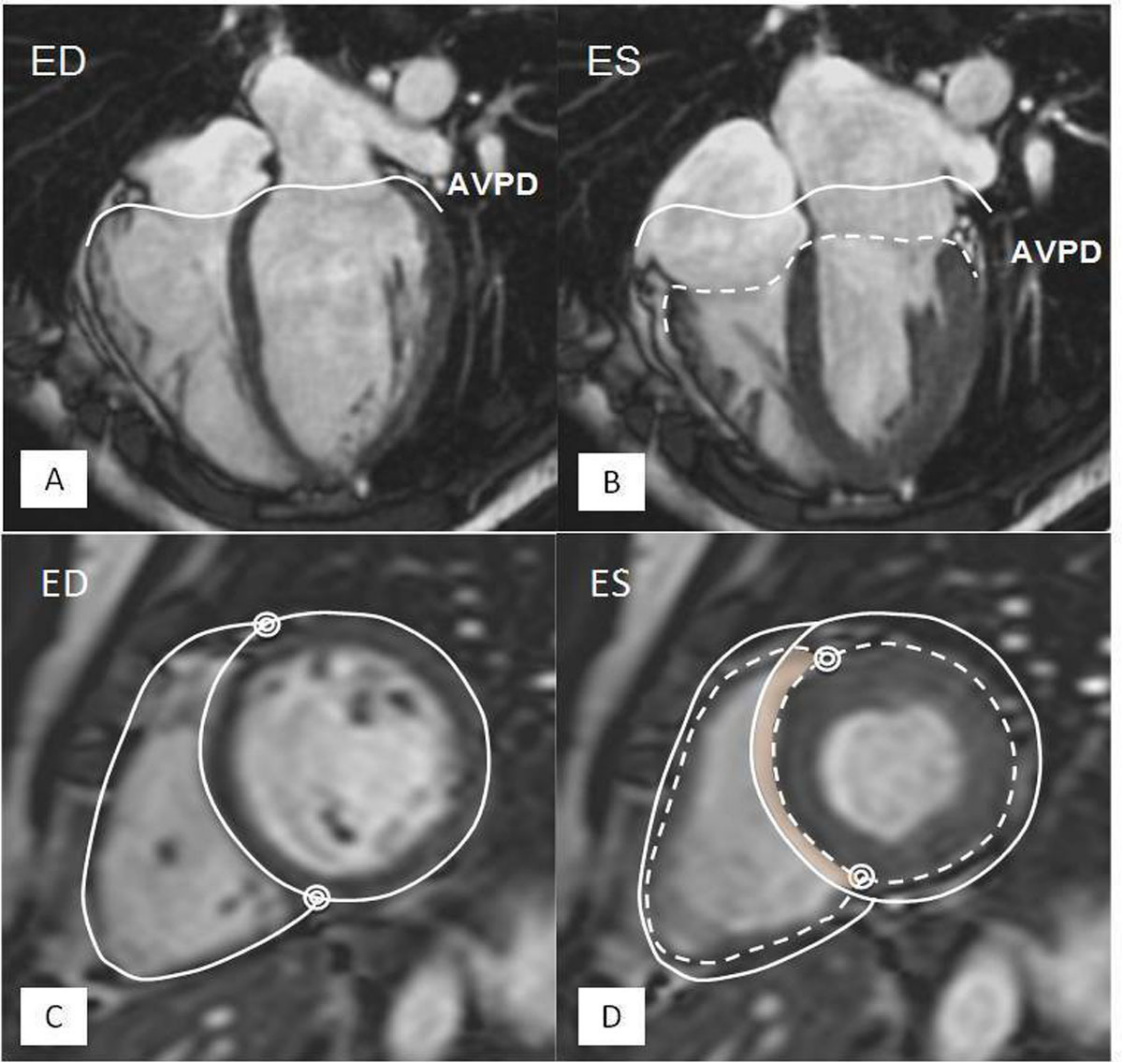
Longitudinal contribution (top) with lateral and septal movement demonstrated (bottom) in end diastole (ED) and in end systole (ES). The longitudinal function, calculated from the atrioventricular plane displacement (AVPD) is demonstrated in 4 chamber view. The volume derived from the AVPD is the area between the full and dashed outline of the AVPD. The lateral and septal movement is demonstrated in short axis view. The volume derived from the lateral movement is the area between the full and dashed outline of the outer delineation. The right ventricular insertion points are marked with double circles. The faint colored areas are the septal contribution to stroke volume, in this case moving to the left in systole

Twenty patients (11 females) evaluated with right heart catheterization (RHC) for pulmonary hypertension were studied and CMR was used for assessment of cardiac volumes. Longitudinal function of the RV and LV was calculated from atrioventricular plane displacement (AVPD) in long-axis views and area between end-diastole and end-systole (Figure 1). Lateral and septal movement was calculated from short axis stack (Figure 1). Volume was derived from the lateral movement between the end-diastolic and end-systolic outline of the outer delineation of the ventricles. Right ventricular insertion points were marked and used to calculate septal contribution to stroke volume. Septal motion towards LV was denoted as positive contribution to LV SV.

Thirty-three healthy adult volunteers (13 females) were used as controls for volumes.

## Results

**Figure 2:**
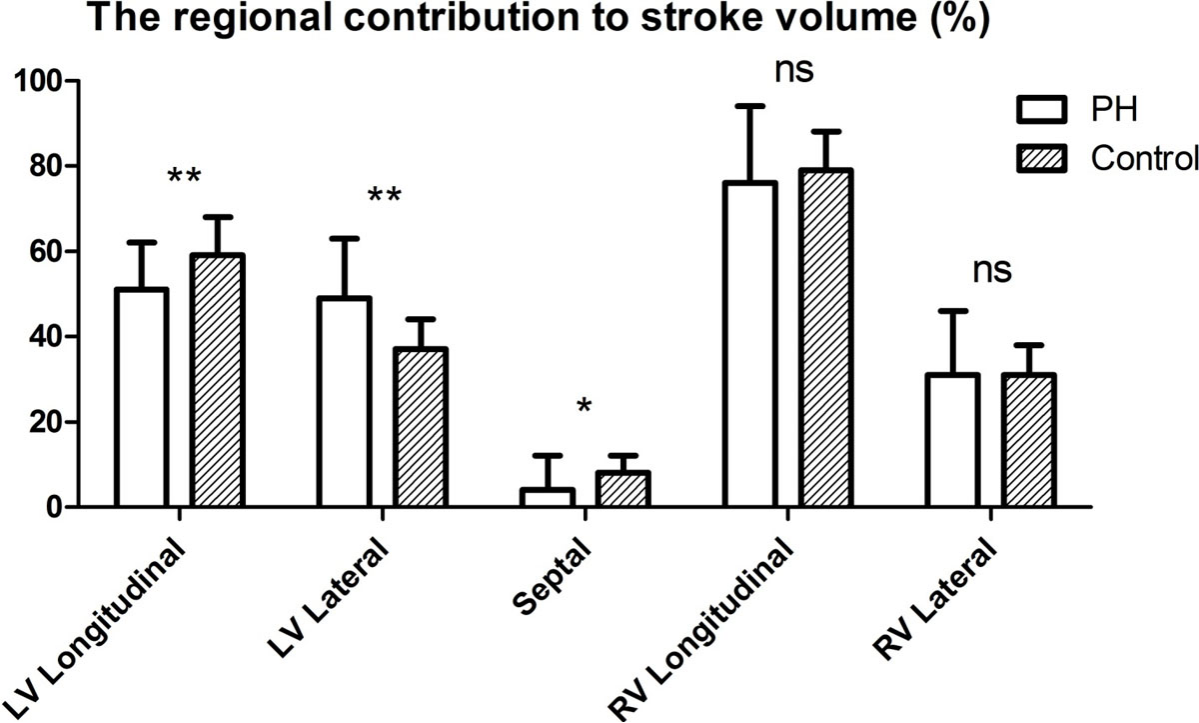
The relative contribution of longitudinal, septal and lateral contraction to stroke volume of the left (LV) and right ventricle (RV) in pulmonary hypertension (PH) and normal controls.

Systolic and mean pulmonary arterial pressure (sPAP and mPAP) in PH patients were 75±25mmHg and 47±16mmHg with LV and RV ejection fraction of 56±11% and 33±12%, respectively. AVPD in patients were smaller (10.9±3.1mm for LV and 12.2±3.7mm for RV) than in controls (16.6±1.9mm for LV and 21.8±2.2mm for RV, p<0.0001 for both). However, longitudinal contribution to RV SV was not different in patients from healthy subjects; explained by larger RV area in patients compared to controls. Regional contribution to RV and LV SV are shown in figure 2. On the right side, there was a positive correlation between septal and longitudinal contribution to SV in patients (r=0.594, p<0.01) and an inverse correlation between lateral and longitudinal contribution to SV (r=-0.726, p<0.001). The latter correlation was not seen in healthy subjects and not on the left side in patients (ns). There were no significant correlations between sPAP and longitudinal, septal or lateral contributions to RV and LV SV (ns).

## Conclusions

LV pumping in PH patients is affected with decreased longitudinal and increased lateral contribution to LV SV even with normal ejection fraction. In the RV, however, longitudinal and lateral contributions to RV SV are preserved in PH patients. There is a wide range of septal contribution to SV in PH patients. The prognostic implications remains to be determined.

## Funding

The Swedish Research Council, the Swedish Heart-Lung Foundation, the Medical Faculty of Lund University, and the Region of Skåne.

